# Hydrogen Sulfide Effects on the Survival of Lactobacilli with Emphasis on the Development of Inflammatory Bowel Diseases

**DOI:** 10.3390/biom9120752

**Published:** 2019-11-20

**Authors:** Ivan Kushkevych, Věra Kotrsová, Dani Dordević, Leona Buňková, Monika Vítězová, Amedeo Amedei

**Affiliations:** 1 Department of Experimental Biology, Faculty of Science, Masaryk University, Kamenice 753/5, 62500 Brno, Czech Republic; vera.kotrsova@ceitec.muni.cz (V.K.); vitezova@sci.muni.cz (M.V.); 2 Department of Plant Origin Foodstuffs Hygiene and Technology, Faculty of Veterinary Hygiene and Ecology, University of Veterinary and Pharmaceutical Sciences, 61242 Brno, Czech Republic; dani_dordevic@yahoo.com; 3 The Department of Environmental Protection Engineering, Faculty of Technology, Tomas Bata University in Zlín, 76001 Zlín, Czech Republic; bunkova@utb.cz; 4 Department of Experimental and Clinical Medicine, University of Florence, 50134 Florence, Italy; amedeo.amedei@unifi.it

**Keywords:** hydrogen sulfide, toxicity, intestinal microbiome, sulfate-reducing bacteria, lactic acid bacteria, inflammatory bowel disease, ulcerative colitis

## Abstract

The gut microbiota is a complex component of humans that depends on diet, host genome, and lifestyle. The background: The study purpose is to find relations between nutrition, intestinal lactic acid bacteria (LAB) from various environments (human, animal intestine, and yogurt) and sulfate-reducing microbial communities in the large intestine; to compare kinetic growth parameters of LAB; and to determine their sensitivity to different concentration of hydrogen sulfide produced by intestinal sulfate-reducing bacteria. Methods: Microbiological (isolation and identification), biochemical (electrophoresis), molecular biology methods (DNA isolation and PCR analysis), and statistical processing (average and standard error calculations) of the results were used. The results: The toxicity of hydrogen sulfide produced by sulfate-reducing bacteria, the survival of lactic acid bacteria, and minimal inhibitory concentrations (MIC) were determined. The measured hydrogen sulfide sensitivity values were the same for *L. paracasei* and *L. reuteri* (MIC > 1.1 mM). In addition, *L. plantarum* and *L.* *fermentum* showed also a similar sensitivity (MIC > 0.45 mM) but significantly (*p* < 0.05) lower than *L.* *reuteri* and *L. paracasei* (1.1 > 0.45 mM). *L. paracasei* and *L. reuteri* are more sensitive to hydrogen sulfide than *L. fermentum* and *L. plantarum*. *L. pentosus* was sensitive to the extremely low concentration of H_2_S (MIC > 0.15 mM). Conclusions: The *Lactobacillus* species were significantly sensitive to hydrogen sulfide, which is a final metabolite of intestinal sulfate-reducing bacteria. The results are definitely helpful for a better understanding of complicated interaction among intestinal microbiota and nutrition.

## 1. Introduction

One of the main goals of the World Health Organization is the treatment of malnutrition among children under 5 years old. Protective factors against malnutrition are breastfeeding, food, and water safety, the same as these factors play an important role in healthy gut microbiota (GM) [1]. The gut microbiota is a complex component of humans that depends on diet [2], host genome, and lifestyle. Increasing data suggest that the GM modulates several host pathways, playing a key role in human physiology and impacting in the development of different pathologic disorders, such as inflammatory bowel diseases (IBD), obesity [3], autism spectrum disorders, stroke, and cancer, especially colorectal cancer [4].

The typical maternal probiotic is lactic acid bacteria (LAB), and the absence of these bacteria leads to inefficient absorption of macro and micronutrients from food, while their absence is associated with diarrhea and pathogens invasion. Studies have started to focus on trials including non-toxic missing microbes and nutrients necessary to restore LAB and healthy mature anaerobic gut microbiota [1]. The proper functioning of the digestive tract is secured by the LAB presence since lactic acid bacteria represent an integral GM component. The main LABs include *Lactococcus* sp., *Bifidobacterium* sp., *Lactobacillus* sp., *Streptococcus* sp., *Leuconostoc* sp., *Pediococcus* sp., and *Enterococcus* sp. [5]. This heterogeneous group of bacteria can be found in various environments including human, animals, and plants. It has been confirmed that some probiotic strains have health benefits as they prevent bacterial translocation and gut infections [6].

The final product of LAB fermentative metabolism is lactic acid [7]. Homofermetative LABs are *Lactobacillus acidophilus*, *Streptococcus salivarius*, *Lactococcus lactis*; heterofermentative LABs are *Lactobacillus brevis*, *Lactobacillus fermentum*, and *Leuconostoc mesenteroides*. The heterofermentative products are lactic acid, acetic acid, ethanol, and carbon dioxide [8,9]. Since LAB belong to an acidophilic group, their cultivation has to be done by a medium that includes carbohydrates, amino acids, peptides, vitamins, and nucleic acid derivatives [10].

Different studies observed reduced LAB levels in subjects with inflammatory bowel disease, in particular, ulcerative colitis (UC) [11,12]. However, the main issue in a UC explanation is still the unclear causes of its development. At present, it is explained as a combination of environmental and genetic factors. On the other side, numerous studies have found a certain relation between the occurrence of sulfate-reducing bacteria (SRB), their hydrogen sulfide overproduction, and the UC occurrence [13,14,15,16,17]. Toxic hydrogen sulfide is the final SRB metabolic product since they use sulfate as an electron acceptor [18,19]. Sulfate intake is highly dependent on diet, due to its occurrence in the following food commodities: some breads, dried fruits, brassicas, sausages, some beers, ciders, and wines [20]. The whole intestinal microbiome is under the influence of diet, host lifestyle, chemotherapeutic treatment, similar to the complicated relationship between LAB and microorganisms in the intestines [21]. The fluctuations in the gut microbiome can be a trigger and the cause of IBD, such as UC. Studies that include GM investigation and monitoring are very important since it has been confirmed that processes in the intestinal microbiome play an important role in different physiological processes existing in human and animal bodies.

In recent years, there are many types of research dedicated to the antibiotic sensitivity of LAB since these microorganisms are most sensitive to antibiotics and chemotherapy. However, inhibitory concentrations and the mechanism of action of hydrogen sulfide (produced by SRB) on LAB has never been reported and studied before.

The purpose of the study is to find relations between nutrition, intestinal lactic acid bacteria from various environments (human, animal intestine, and yogurt), and sulfate-reducing microbial communities in the large intestine; to compare the kinetic growth parameters of LAB, and to determine their sensitivity to different concentrations of hydrogen sulfide produced by intestinal SRB.

## 2. Materials and Methods

### 2.1. Isolation and Identification of Lactic Acid Bacteria

MRS medium (Sigma-Aldrich, Prague, Czech Republic) was used for the isolation of five LAB species from different environments. In the samples of human and mice feces, yogurt, similar to probiotic pills, were added to the tube with MRS medium, and they were cultured for 24 h at 37 °C in a thermostat. The mixed cultures were diluted, after 24 h, and plated on Petri dishes (MRS agar). Chosen bacterial colonies were isolated and purified using the streak plate method and stored in the fridge. LAB strains were kept at the Laboratory of Anaerobic Microorganisms of the Department of Experimental Biology at Masaryk University (Brno, Czech Republic). The first step for bacterial identification was microscopic methods. The bacterial cultures were fixed to the microscope slide using a fire flame and then treated with Gram staining. Distilled water was used between each step to wash out a dye residue. The bacterial strains were subjected to a microscopic analysis at 1000× magnification using a light microscope (Olympus BX50, Olympus Czech Group, Prague, Czech Republic).

### 2.2. DNA Isolation and Polymerase Chain Reaction (PCR)

A commercial kit QIAmp DNA Mini Kit (QIAGEN, catalog number 51304) was used for DNA isolation. Bacterial samples were identified by 16S rRNA gene sequencing with primers chosen according to the Weisburg et al., 1991 [22]:

8FPL 5′- AGT TTG ATC CTG GCT CAG - 3´

806R 5′ - GGT TAC CTT GTT ACG ACT T - 3´

Obtained DNA lysates were prepared for the amplification. PCR mixture was prepared by the following protocol: MasterMix (100 µL), primer 8FPL (100 μM)—1.0 µL, primer 806R (100 μM)—1.0 µL, uracil-DNA glycosylase—1.0 µL, deionized water—77.0 µL. There was 180 µL of PCR mixture, in total. Of the PCR mixture, 18 µL was added to the PCR tube. Of DNA lysate, 2 µL was added.

### 2.3. The Sequence Analysis of 16S rRNA

Purified amplicons were sequenced, and obtained sequences were compared with known reference strains in the database of the National Center for Biotechnology Information (NCBI) using the basic local alignment search tool (BLAST). The unique ID numbers for each sequence were obtained and saved in GenBank (*Lactobacillus pentosus* MK736277, *L. paracasei* MK736278, *L. plantarum* MK736279, *L. fermentum* MK736280, and *L. reuteri* MK736281).

### 2.4. Electrophoresis

Warmed up agarose gel of 1.5%, including 55 µL of GelRed (Sigma-Aldrich, Prague, Czech Republic), was poured to the electrophoresis tray. Loading dye of 0.5 µL and 3.5 µL of water were mixed with 1 µL of the sample. Three µL of the ladder and 5 µL of the sample mixtures were loaded to the agarose gel. Electrophoresis was conducted for 60 min in a Tris-borate-EDTA (TBE) buffer. Agarose gel was run at a voltage of 80 V. The gel was analyzed by G: BOX F3-Gel Imaging (Figure S1). Five µL of primer 8FPL was added to each tube with 5 µL of the amplified sample.

### 2.5. Bacterial Growth Parameters Determination

Growth parameters of each strain were calculated using an optical density measurement, and growth curves were created by a Bioscreen C spectrophotometer (Dynex Technologies, Prague, Czech Republic) that included CFU calculation, same as calibration curve creation. The values included in the calculations of individual curves were selected in accordance with the scheme (Figure S2). The growth parameter calculations were done by the following formulas.

Average division rate (R): the number of generations related to the growth time of the population (*t* = time; *X* = colony forming units per milliliter):

$$R=\frac{1}{Log2}\;\cdot \;\frac{LogX-LogX_{0}}{t-t_{0}}$$

Generation time (τ): the time required to form one generation of cells (the time between two divisions):

$$\tau =Log2\cdot \frac{t-t_{0}\;}{LogX-LogX_{0}}$$

Specific growth rate (µ): means the growth rate per unit of the CFU or biomass:

$$\mu =2.3\;\cdot \;\frac{LogX-LogX_{0}}{t-t_{0}}$$

Lag time (L): time of the Lag phase where the cells adapt to the environment and the necessary substances are synthesized:

$$L=t_{k}-t_{e}$$

*t_k_*: time from the start of the experiment to the time of the end of the stationary phase

*t_e_*: time of exponential phase, which can be calculated by the formula:

$$t_{e}=\frac{1}{Log2}\cdot \tau \cdot \left(LogX-LogX_{0}\right)$$

### 2.6. The Evaluation of Minimal Inhibitory Concentrations (MIC)

Sodium sulfide was used as the source of the hydrogen sulfide. LAB cultures were cultivated during a 24 h period in the presence of hydrogen sulfide (Figure S3). One hundred μL of Na_2_S of various concentrations (from 0.038 to 1.5 mM) were added to the tubes containing 2 mL of MRS media. The media was inoculated with 24 h of LAB cultivation; control samples did not contain Na_2_S. The spectrophotometric method was used for the determination of samples’ optical density. Twenty-five μL of the sample diluted by a serial dilution was added on ¼ of the MRS agar plate, and the counting of CFU was done after 24 h.

### 2.7. Statistical Analysis

The main statistical parameters (M—mean, S_E_—standard error, M ± S_E_) based on the experimental data were calculated [23]. Statistical significant (*p* < 0.05) differences were measured by principal component analysis (PCA) and cluster analysis. The statistical analysis was carried out by SPSS 20 statistical software (IBM Corporation, Armonk, NY, USA). The plots were built by software package Origin7.0 (Northampton, MA, USA).

## 3. Results

**Figure 1:**
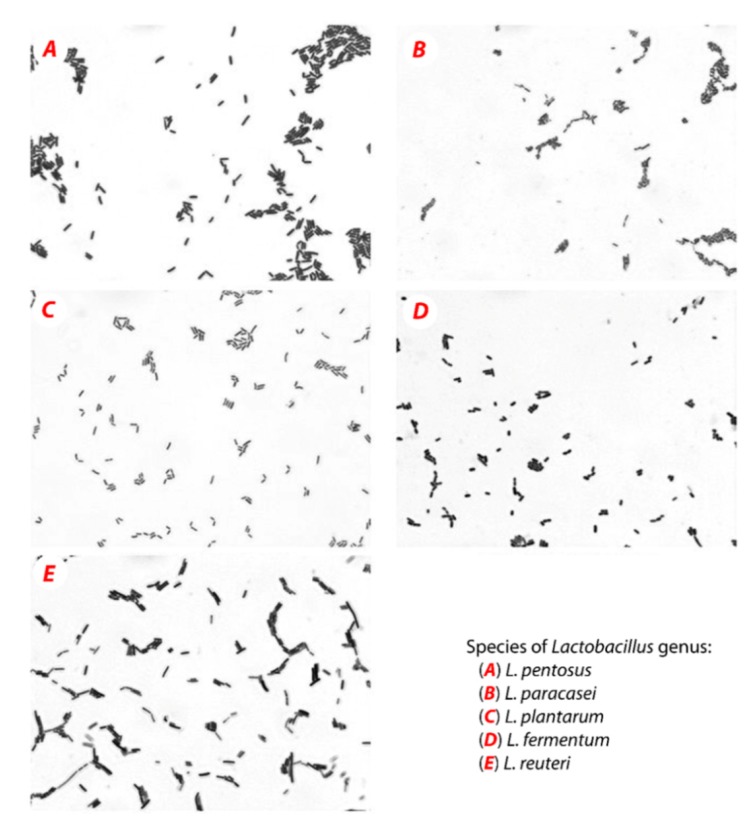
Pure cultures of LAB cells (light microscope, magnification 1000×).

The results are shown in Figure 1. All strains belonged to the *Lactobacillus* genus, and this was confirmed by sequence analysis of 16S rRNA of LAB cultures, isolated from various environments. Nucleotide sequences were identified (Table 1), according to the National Center for Biotechnology Information and the basic local alignment search tool.

**Figure 2:**
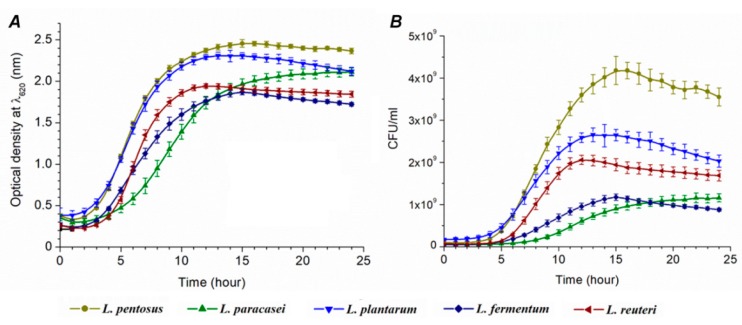
Growth curves of lactic acid bacteria (M ± S_E_, *n* = 5): data gained by the Bioscreen C spectrophotometer (**A**) and designed by CFU values (**B**).

The optical density was measured for 24 h (Supplement Table S1), and the growth curve shown in Figure 2A was constructed. OD values were used for better orientation and understanding, the same as for the comparison with CFU growth curves (Figure 2B). The calibration curves were used for the conversion of OD to CFU (Table 2 and Supplement Figure S4). CFU value-conditioned growth curves are shown in Supplement Table S2 and Figure 2B. The following growth parameters were calculated: average division ratio, specific growth rate, generation time, and lag time.

The resulting growth parameters are presented in Table 2. The smallest *R* value was observed for *L. paracasei* (0.243 h^−1^) and the highest for *L. reuteri* (0.650 h^−1^). It means that *L. paracasei* needs the shortest time period for cell division in comparison with other species. Conversely, *L. reuteri* needs the longest time. Significant differences cannot be observed among other species. The longest τ (generation time) was estimated for *L. paracasei* (4.151 h) and the shortest one for *L. reuteri* (1.539 h).

**Figure 3:**
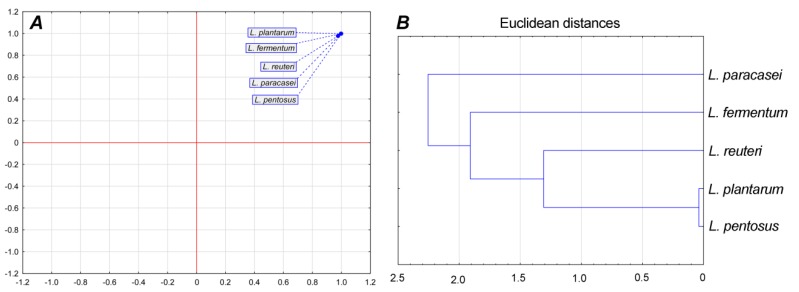
Principal component analysis of growth parameters (***A***) and cluster analysis (***B***).

Principal component analysis showed that there were no statistically significant (*p* < 0.05) differences between LAB isolates in overall parameters (average generation time, division ratio, and specific growth rate) since only one group can be observed in Figure 3*A*. Cluster analysis showed that *L. plantarum* and *L. pentosus* belong to one cluster; they were the most similar according to measured physiological parameters (Figure 3*B*).

**Figure 4:**
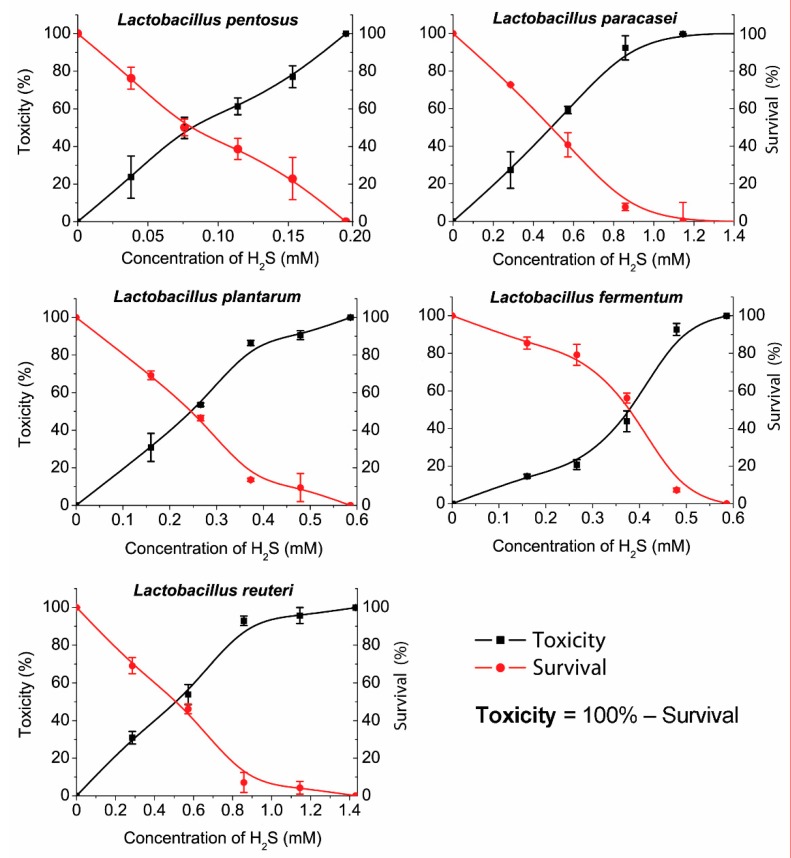
The survival of *Lactobacillus* species and the toxicity of hydrogen sulfide.

The trend of µ values (specific growth rate) is in accordance to the trend of R values where the lowest µ value was observed for *L. paracasei* (0.168 h) and the highest for *L. reuteri* (0.450 h). The longest lag time (9 h) was measured for *L. paracasei* that is in relation to the highest generation time (4.151 h) within the same species (Supplement Table S4). The percentage of hydrogen sulfide toxicity toward bacterial cells, the same as the percentage of viable cells, is shown in Figure 4.

Different sensitivity levels were measured among the different LAB species. Toxicity, survival, and MIC were also determined. The measured hydrogen sulfide sensitivity values were the same for *L. reuteri* and *L. paracasei* (MIC > 1.1 mM). Also, *L. fermentum* and *L. plantarum* showed a similar sensitivity (MIC > 0.45 mM) but significantly lower than *L. reuteri* and *L. paracasei* (1.1 > 0.45 mM). *L. reuteri* and *L. paracasei* are more sensitive to a hydrogen sulfide presence than *L. fermentum* and *L. plantarum*. *L. pentosus* was inhibited at very low concentrations of H_2_S (MIC > 0.15 mM). According to the graph (Figure 3), the toxicity and viability trends of *L. pentosus* are almost linear, especially in comparison with other species that have shown exponential trends of toxicity and viability. Notably, the data showed that *L. pentosus* is the most sensitive to the presence of hydrogen sulfide.

The IC_50_ values that indicate 50% of dead (or viable) bacteria in the presence of hydrogen sulfide can also be noticed out of plotted graphs. For *L. reuteri* and *L. paracasei*, the IC_50_ is almost the same (IC_50_ > 0.45 mM). *L. fermentum* and *L. plantarum* had similar MIC values, but their IC_50_ differ significantly (*L. fermentum* >0.4 mM; *L. plantarum* >0.25 mM). Out of our results, it can be overviewed that all species had a significant trend in relative toxicity growth, similar to the decrease in bacterial survival, though the results obtained for *L. fermentum* can be considered exceptional.

Isolated LAB from different sources (human feces, probiotic supplement, yogurt, mice feces) had physiologically similar properties, but they have different sensitivity toward hydrogen sulfide.

## 4. Discussion

Lactic acid bacteria are undoubtedly forming an important part of gut microbiota, and proper functioning of the digestive tract depends on their presence. The counts of lactic acid bacteria are significantly reduced in the presence of SRB since they produce toxic hydrogen sulfide. The importance of gut microbiota and the roles of SRB and LAB have been constantly dealt with by many previous studies [24,25]. At the same time, commercially produced probiotic supplements mainly consist of the genus *Lactobacillus*. The members of the genus *Lactobacillus* are probably one of the most studied and understood LAB (Heeney et al. (2018)) [26]. The ideal way for this species’ isolation is from the host’s stool, though they could be significantly changed by the environment of the host’s digestive tract. Human feces (*L. pentosus, L. paracasei*), probiotic supplements (*L. plantarum*), mice feces (*L. reuteri*), and yogurt (*L. fermentum*) were environments used in the isolation research. LAB counts are noticeably reduced during inflammatory bowel diseases, including ulcerative colitis, while the numbers of SRB increase; thus, it resulted in higher H_2_S concentrations in the intestines.

Our results reveal information about how H_2_S affects lag phase (*L*) extension and the time of cell division (τ). The kinetic parameters of *Desulfovibrio piger* Vib-7 to H_2_S were tested in our previous study, indicating that lag phase is doubled in the presence of increased concentrations of H_2_S, while the generation time was extended eight times [18]. Particularly in *L. paracasei*, in both liquid and solid media, a longer growth time was noticed. *L. acidophillus* OSU133 growth parameters were tested by Cho et al. (1996) [27]. In their study, the generation time of the strain ranged from 0.3 to 1.2 h and the lag phase from 1.2 to 6.9 h. Similar results (1.2–1.4 h) were observed by Brizuela et al. (2001) [28]. Gorbach and Goldin (1991) found that the generation time of lactobacilli was one hour [29]. OD and CFU growth curves that differed in terms of growth tendency represent an interesting finding of our study. The increase of microbial biomass indicates that the trend of the growth between species is rather similar. *L. pentosus* had the largest increase in the biomass for 24 h (cultivation in tubes and agar plates), while *L. paracasei* had the smallest one.

The focus of our study was to find the relationship between different concentrations of hydrogen sulfide and lactic acid bacteria (*Lactobacillus* genus). Some studies also suggest the positive effect of H_2_S on macro- and microorganisms [18,19,30,31]. H_2_S produced by bacteria was also found to serve as the defense against antimicrobial compounds, indicating that the cytoprotective effect of H_2_S is probably a universal mechanism of defense from bacteria to mammals. Reis et al. (1992) found that SRBs are completely inhibited when they are exposed to H_2_S concentrations higher than 16.1 mM [32]. UC environment is reflected as an increased SRB number and a consequently higher concentration of H_2_S. SRBs are not the only species in the intestines capable of producing H_2_S. The following bacteria can also secrete H_2_S: *Clostridium*, *Escherichia*, *Salmonella*, *Fusobacterium*, *Klebsiella*, *Desulfovibrio*, and *Enterobacter* [33]. Clostridia, bifidobacteria, and the *Bacteroides fragilis* group can degrade sulfated substance, such as colonic mucin, and release free sulfate that can be utilized by SRBs (Gibson et al., 1993) [11]. SRBs can use sulfate as an electron acceptor, an increase in the numbers, to make an intestinal environment appropriate for IBD development.

The important factors affecting the intestinal environment are sulfate consumption, sulfide production, lactate consumption, and acetate accumulation [34,35,36,37,38,39,40]. The *Desulfovibrio* genus is very often present in the intestines and feces of humans and animals with IBD [41,42,43,44]. These bacteria in their metabolism use sulfate (terminal electron acceptor) and organic compounds that serve as electron donors [45,46,47]. Our previous study indicated that *Desulfovibrio* strains from individuals with colitis were grouped in one cluster by biomass accumulation and sulfide production, and another cluster was formed by the strains from healthy individuals. Acetate produced by SRB can also be in synergic interaction with H_2_S, though lactate oxidation represents only minor factors in bowel disease [34]. These conditions may be one of the main UC causes that may lead to a higher incidence of bowel cancer [48,49,50]. Hydrogen sulfide adversely affects intestinal mucosa and epithelial cells, causes phagocytosis, inhibits the growth of colonocytes, causes the death of intestinal bacteria, and induces hyperproliferation and metabolic abnormalities of epithelial cells [31,51]. The SRB presence is also connected with colon inflammation. Therefore, the integrity of colonocytes depends on hydrogen sulfide concentration [42,48,49].

Other research describing cross-correlation parameters of the SRB metabolic process found out that the strains isolated from people with colitis shifted to the right side of the Y-axis by biomass accumulation, sulfate consumption, lactate oxidation, as well as hydrogen sulfide and acetate production, in comparison with the strains from healthy individuals [34]. It should be stressed that the gut microbiota is a very complex matrix (interactions with clostridia, methanogens, lactic acid bacteria, etc.) and due to it, our study can be limited [41,52,53]. Otherwise, it can also be emphasized that a central role in the development of IBD is SRB [31,42,43,44,54]. Since this bacterial group produces hydrogen sulfide, it can inhibit other microbiota, such as lactic acid bacteria, methanogens, similar to many other intestinal microorganisms [41,54].

A diet high in sulfate ions (preservatives added to food often contain sulfur oxides) leads to an increase in hydrogen sulfide concentration by SRB in rumens. The Western diet contains over 16.6 mmol sulfate/day [20], feces of healthy individuals (approximately 50%) contain SRB (up to 92% belong to *Desulfovibrio genus*) [11,36]. Sulfate polysaccharides such as mucin, chondroitin sulfate, and carrageenan are widely consumed, and they are also good sources of sulfate for SRB [31]. Hydrogen sulfide can also be toxic for its producers. The high toxicity of H_2_S was measured at concentrations higher than 6 mM; the growth was stopped, but 100% inhibition of metabolic activities was not achieved [19].

Attention must also be paid to the fact that the beneficial effect of hydrogen sulfide formed in the intestine on the general condition of the body, e.g., regulation of arterial pressure, has not been taken into account [55,56]. Similarly, the beneficial H_2_S role in inflammation, which can act as an antioxidant [57].

The species used in industry, such as LAB for yogurt production, are thought to be more resistant to adverse environments. *L. plantarum* (probiotic supplement) and *L. fermentum* (yogurt) showed almost the same sensitivity (MIC > 0.45 mM). It was also perceived that species isolated from the human intestine (from healthy individuals) are more sensitive to H_2_S, but the results of the research showed this in *L. pentosus* (MIC > 0.15 mM) isolated from the human feces. Oppositely, *L. paracasei* had a lesser sensitivity to H_2_S exposure (MIC > 1.1 mM) than *L. plantarum* (from the probiotic supplement) (MIC > 0.45 mM), meaning that the assumption mentioned above is rejected. The level of hydrogen sulfide in the fecal matter of a healthy human adult ranges from 0.3 to 3.4 mmol/l [31]. Some areas may have a higher concentration, while others may have lower. The MIC experiment indicates that the level of H_2_S even in the healthy intestine can be enough for the inhibition of the tested species, especially *L. pentosus*. The values reported in the publication were measured under conditions that are similar to the intestinal environment (a variety of substrates, hundreds of different bacterial species, the same as bacterial and host metabolites, etc.). It is possible that bacterial species other than lactobacillus may prevail in higher H_2_S levels while lactobacillus may prevail at lower H_2_S levels, thus striking a balance. It should be noted that the intestinal microbiota is a very complex system. This research was focused on in an in vitro test with pure cultures of *Lactobacillus* species only. However, among lactic acid bacteria, the *Lactobacillus* species are not alone in the intestinal tract. There are also *Bifidobacterium*, *Lactococcus*, *Streptococcus*, and other lactic acid bacterial genera, which are also no less important in the gut. Certainly, future studies should also include a mixed culture in vitro as well as in vivo. These conditions would more simulate the intestinal environment. Also, LAB strains should be tested by binding a specific probe and evaluated by cytometer flow.

## 5. Conclusions

It is well known that lactic acid bacteria represent a beneficial factor for the host organism. These bacteria, especially their final product of metabolism (lactic acid), play an important role in food fermentation; they fight against pathogenic microorganisms and represent an important element for the whole intestinal ecosystem. Antibiotics or metabolites of other bacteria have an inhibitory effect on LAB. Research has shown that *L. pentosus* (from human feces) has the highest sensitivity to H_2_S. Conversely, *L. paracasei* (isolated from human feces) were the most resistant bacteria among other identified *Lactobacillus* species: *L. fermentum* (yogurt), *L. plantarum* (probiotic supplement), and *L. reuteri* (mice feces). The *Lactobacillus* species showed significant sensitivity toward hydrogen sulfide. Certainly, the findings of the study will be helpful in future experiments including processes around the intestinal environment affected by inflammatory diseases. The research gives a broader picture of the potentially inhibitory environment toward the presence of health-beneficial LAB strains. Our data can also be connected with previous studies that have found a relationship between reduced numbers of LAB in the presence of *Desulfovibrio* bacteria leading to higher prevalence of IBD. Especially, the occurrence of ulcer is connected with lower LAB counts that can serve as a signal of processes in the intestines. These processes could be indicators for serious ailments of humans and animals.

## Figures and Tables

**Table 1:** Lactic acid bacteria (LAB) sequence analysis of 16S rRNA.

Sources of Isolation.	Species	ID Number in GenBank
Human feces 1	*Lactobacillus pentosus*	MK736277
Human feces 2	*Lactobacillus paracasei*	MK736278
Probiotic supplement	*Lactobacillus plantarum*	MK736279
Yogurt	*Lactobacillus fermentum*	MK736280
Mice feces	*Lactobacillus reuteri*	MK736281

**Table 2:** The average generation time(τ), division ratio (R), lag time (L), and specific growth rate (μ).

Isolates of LAB	R (h^−1^)	τ (h)	μ (h)	*L* (h)
*L. pentosus*	0.428 ± 0.0034	2.338 ± 0.0188	0.296 ± 0.0024	5
*L. paracasei*	0.243 ±0.0230	4.151 ± 0.3460	0.168 ±0.0159	9
*L. plantarum*	0.421 ± 0.0118	2.375 ± 0.0664	0.292 ±0.0082	5
*L. fermentum*	0.322 ± 0.0141	3.116 ± 0.1310	0.223 ± 0.0098	7
*L. reuteri*	0.650 ± 0.0172	1.539 ± 0.0420	0.450 ± 0.0119	6

## Supplementary Materials

The following are available online at https://www.mdpi.com/2218-273X/9/12/752/s1, Figure S1: Electrophoresis gel: samples (1–5), negative control (NC). Bands are in accordance with the presence of the amplified DNA; Figure S2: The typical bacterial growth curve: Log X, Log X_0_, t and t_0_ mark location of the values for the bacterial growth parameters; t_k_—the time of the experiment, t_e_—the time of exponential phase; L is the time of lag phase; Figure S3: LAB cultivation (24 h) under the influence of H_2_S. After 24 h the optical density (OD) was determined and, consequently, the reacting mixture was diluted and spread on the MRS agar; Figure S4: The calibration curves used for the conversion of CFU to OD; Table S1: Optical density data measured by Bioscreen C spectrophotometer (M ± S_E_, *n* = 5); Table S2: The conversion of OD to CFU used to create the calibration curves for *Lactobacillus* species; Table S3: CFU values calculated by calibration curves equations (M ± S_E_, *n* = 5); Table S4: The minimal inhibitory concentrations for *Lactobacillus* species.
