# Supplementary material for: Hydrogen Sulfide Effects on the Survival of Lactobacilli with Emphasis on the Development of Inflammatory Bowel Diseases
Source: Biomolecules. 2019 Nov 20;9(12):752. doi: 10.3390/biom9120752 (PMC6995546; doi:10.3390/biom9120752)
Supplement: Supplementary file 1 [file biomolecules-09-00752-s001.pdf]

Supplementary

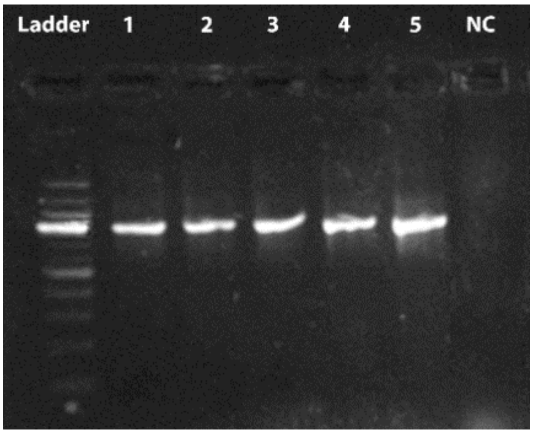

Figure 1. Electrophoresis gel: samples (1–5), negative control (NC). Bands are in accordance with the presence of the amplified DNA.

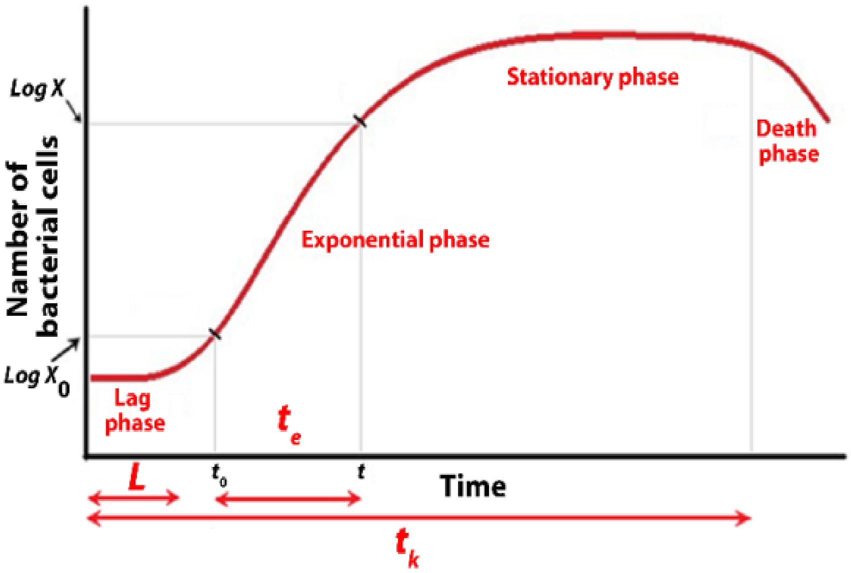

Figure 2. The typical bacterial growth curve: *Log X*, *Log X_0*, *t* and t_0 mark location of the values for the bacterial growth parameters; t_k – the time of the experiment, t_e – the time of exponential phase.

L – is the time of lag phase.

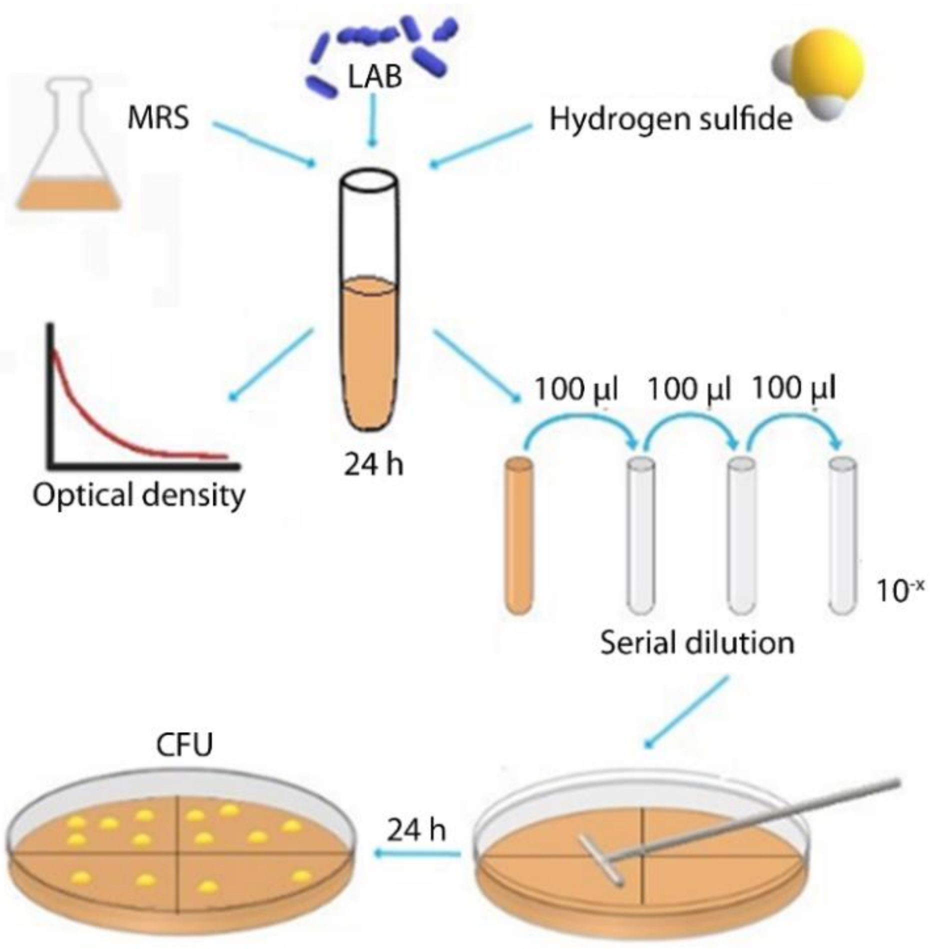

Figure 3. LAB cultivation (24 h) under the influence of H_2S. After 24 h the optical density (OD) was determined and, consequently, the reacting mixture was diluted and spread on the MRS agar.

Table 1. Optical density data measured by Bioscreen C spectrophotometer (M ± S_E, n = 5).

Time (hour)	*L. Pentosus*		*L. Paracasei*		*L. Plantarum*		*L. Fermentum*		*L. Reuteri*	
	**OD**	**SDEV**	**OD**	**SDEV**	**OD**	**SDEV**	**OD**	**SDEV**	**OD**	**SDEV**
0	0.380	0.0236	0.354	0.0338	0.385	0.0883	0.265	0.0166	0.267	0.0237
1	0.322	0.0080	0.295	0.0398	0.382	0.0574	0.234	0.0060	0.225	0.0121
2	0.358	0.0101	0.305	0.0389	0.422	0.0467	0.254	0.0076	0.228	0.0108
3	0.469	0.0132	0.338	0.0426	0.533	0.0500	0.316	0.0118	0.267	0.0152
4	0.699	0.0183	0.393	0.0469	0.741	0.0506	0.463	0.0232	0.358	0.0236
5	1.095	0.0292	0.471	0.0555	1.058	0.0360	0.685	0.0374	0.568	0.0421
6	1.487	0.0310	0.583	0.0691	1.431	0.0688	0.927	0.0502	0.972	0.0588
7	1.797	0.0317	0.740	0.0933	1.722	0.0831	1.129	0.0630	1.353	0.0652
8	2.003	0.0310	0.949	0.0976	1.937	0.0628	1.331	0.0742	1.595	0.0642
9	2.156	0.0382	1.181	0.0858	2.071	0.0557	1.489	0.0801	1.752	0.0522
10	2.240	0.0348	1.393	0.0890	2.184	0.0460	1.599	0.0692	1.861	0.0397
11	2.321	0.0286	1.592	0.0938	2.248	0.0479	1.698	0.0527	1.923	0.0342
12	2.373	0.0264	1.742	0.0753	2.293	0.0302	1.765	0.0435	1.944	0.0257
13	2.411	0.0340	1.842	0.0654	2.311	0.0422	1.812	0.0412	1.940	0.0304
14	2.434	0.0328	1.913	0.0638	2.305	0.0690	1.851	0.0397	1.929	0.0267
15	2.457	0.0460	1.967	0.0638	2.309	0.0360	1.871	0.0340	1.914	0.0364
16	2.456	0.0268	2.008	0.0588	2.295	0.0359	1.857	0.0257	1.897	0.0366
17	2.446	0.0352	2.036	0.0580	2.278	0.0474	1.834	0.0234	1.893	0.0354

18	2.425	0.0363	2.058	0.0553	2.270	0.0565	1.815	0.0260	1.883	0.0333
19	2.423	0.0232	2.078	0.0579	2.245	0.0410	1.795	0.0225	1.876	0.0381
20	2.401	0.0237	2.090	0.0606	2.215	0.0392	1.781	0.0211	1.871	0.0323
21	2.391	0.0313	2.089	0.0580	2.198	0.0501	1.764	0.0226	1.865	0.0381
22	2.400	0.0204	2.115	0.0589	2.169	0.0385	1.756	0.0244	1.858	0.0342
23	2.386	0.0243	2.105	0.0532	2.145	0.0423	1.740	0.0216	1.848	0.0374
24	2.366	0.0340	2.114	0.0503	2.121	0.0469	1.724	0.0222	1.845	0.0374

Comment: OD = optical density; SDEV = standard deviation

Table 2S. The conversion of OD to CFU used to create the calibration curves for *Lactobacillus* species

OD/CFU	*L. Pentosus*	*L. Paracasei*	*L. Plantarum*	*L. Fermentum*	*L. Reuteri*
OD1	0.279	0.294	0.242	0.365	0.205
CFU1	–	42×10^{7}	–	–	5.1×10^{7}
OD2	0.763	0.595	0.463	0.582	0.551
CFU2	2.1×10^{8}	9.2×10^{7}	2.1×10^{8}	9.6×10^{7}	1.3×10^{8}
OD3	1.216	1.191	1.031	0.815	0.884
CFU3	4.6×10^{8}	1.3×10^{8}	5.2×10^{8}	2.2×10^{8}	2.1×10^{8}
OD4	1.868	1.681	1.869	1.475	1.302
CFU4	6.2×10^{8}	4.4×10^{8}	1.3×10^{9}	5.8×10^{8}	4.2×10^{8}
OD5	2.272	2.174	2.417	1.927	1.879
CFU5	4.8×10^{9}	1.3×10^{9}	3.7×10^{9}	1.2×10^{9}	1.8×10^{9}

Table 3. CFU values calculated by calibration curves equations (M ± S_E, n = 5).

Time (hour)	*L. Pentosus*		*L. Paracasei*		*L. Plantarum*		*L. Fermentum*		*L. Reuteri*	
	CFU	SDEV	CFU	SDEV	CFU	SDEV	CFU	SDEV	CFU	SDEV
0	9.91×10^{7}	4.23×10^{6}	5.53×10^{7}	3.26×10^{6}	1.73×10^{8}	2.36×10^{7}	5.04×10^{7}	1.68×10^{6}	6.87×10^{7}	3.41×10^{6}
1	8.93×10^{7}	1.29×10^{6}	5.00×10^{7}	3.57×10^{6}	1.72×10^{8}	4.59×10^{7}	4.75×10^{7}	5.55×10^{5}	6.32×10^{7}	1.58×10^{6}
2	9.52×10^{7}	1.74×10^{6}	5.08×10^{7}	3.52×10^{6}	1.82×10^{8}	4.47×10^{7}	4.93×10^{7}	7.27×10^{5}	6.35×10^{7}	1.41×10^{6}
3	1.16×10^{8}	2.76×10^{6}	5.38×10^{7}	4.05×10^{6}	2.13×10^{8}	5.36×10^{7}	5.57×10^{7}	1.27×10^{6}	6.87×10^{7}	2.16×10^{6}
4	1.76×10^{8}	5.76×10^{6}	5.92×10^{7}	4.8×10^{6}	2.86×10^{8}	7.23×10^{7}	7.43×10^{7}	3.29×10^{6}	8.27×10^{7}	4.07×10^{6}
5	**3.60×10^{8}**	1.85×10^{7}	6.77×10^{7}	6.38×10^{6}	**4.49×10^{8}**	1.01×10^{8}	1.15×10^{8}	8.11×10^{6}	1.27×10^{8}	1.11×10^{7}
6	7.29×10^{8}	3.97×10^{7}	8.22×10^{7}	9.35×10^{6}	7.62×10^{8}	1.32×10^{8}	1.84×10^{8}	1.72×10^{7}	**2.87×10^{8}**	3.52×10^{7}
7	1.27×10^{9}	7.06×10^{7}	1.08×10^{8}	1.59×10^{7}	1.15×10^{9}	1.49×10^{8}	**2.74×10^{8}**	3.16×10^{7}	6.21×10^{8}	8.32×10^{7}
8	1.85×10^{9}	1.01×10^{8}	1.55×10^{8}	2.78×10^{7}	1.56×10^{9}	1.49×10^{8}	4.07×10^{8}	5.55×10^{7}	1.02×10^{9}	1.32×10^{8}
9	2.43×10^{9}	1.61×10^{8}	**2.31×10^{8}**	4.69×10^{7}	1.89×10^{9}	1.58×10^{8}	5.55×10^{8}	8.21×10^{7}	1.40×10^{9}	1.47×10^{8}
10	2.83×10^{9}	1.70×10^{8}	3.33×10^{8}	6.90×10^{7}	2.22×10^{9}	1.51×10^{8}	6.89×10^{8}	8.82×10^{7}	**1.74×10^{9}**	1.39×10^{8}
11	3.27×10^{9}	1.64×10^{8}	4.70×10^{8}	7.55×10^{7}	**2.43×10^{9}**	1.72×10^{8}	8.37×10^{8}	8.33×10^{7}	1.98×10^{9}	1.36×10^{8}
12	3.60×10^{9}	1.66×10^{8}	6.09×10^{8}	7.38×10^{7}	2.59×10^{9}	1.14×10^{8}	9.53×10^{8}	7.93×10^{7}	2.06×10^{9}	1.08×10^{8}
13	**3.85×10^{9}**	2.28×10^{8}	7.24×10^{8}	7.72×10^{7}	2.66×10^{9}	1.64×10^{8}	**1.04×10^{9}**	8.39×10^{7}	2.04×10^{9}	1.25×10^{8}
14	4.02×10^{9}	2.29×10^{8}	8.18×10^{8}	8.64×10^{7}	2.64×10^{9}	2.66×10^{8}	1.13×10^{9}	8.79×10^{7}	2.00×10^{9}	1.10×10^{8}
15	4.18×10^{9}	3.32×10^{8}	8.99×10^{8}	9.40×10^{7}	2.65×10^{9}	1.36×10^{8}	1.17×10^{9}	7.85×10^{7}	1.94×10^{9}	1.42×10^{8}

16	4.18×10^{9}	1.97×10^{8}	9.63×10^{8}	9.22×10^{7}	2.60×10^{9}	1.36×10^{8}	1.14×10^{9}	5.80×10^{7}	1.87×10^{9}	1.38×10^{8}
17	4.10×10^{9}	2.51×10^{8}	1.01×10^{9}	9.57×10^{7}	2.54×10^{9}	1.78×10^{8}	1.09×10^{9}	5.03×10^{7}	1.86×10^{9}	1.34×10^{8}
18	3.95×10^{9}	2.58×10^{8}	**1.05×10^{9}**	9.57×10^{7}	2.51×10^{9}	2.12×10^{8}	1.05×10^{9}	5.40×10^{7}	1.82×10^{9}	1.24×10^{8}
19	3.94×10^{9}	1.66×10^{8}	1.09×10^{9}	1.04×10^{8}	2.42×10^{9}	1.47×10^{8}	1.01×10^{9}	4.48×10^{7}	1.80×10^{9}	1.43×10^{8}
20	3.78×10^{9}	1.58×10^{8}	1.11×10^{9}	1.11×10^{8}	2.32×10^{9}	1.33×10^{8}	9.83×10^{8}	4.13×10^{7}	1.78×10^{9}	1.16×10^{8}
21	3.72×10^{9}	2.07×10^{8}	1.11×10^{9}	1.06×10^{8}	2.26×10^{9}	1.68×10^{8}	9.51×10^{8}	4.29×10^{7}	1.76×10^{9}	1.37×10^{8}
22	3.78×10^{9}	1.37×10^{8}	1.16×10^{9}	1.12×10^{8}	2.17×10^{9}	1.24×10^{8}	9.36×10^{8}	4.54×10^{7}	1.73×10^{9}	1.22×10^{8}
23	3.68×10^{9}	1.59×10^{8}	1.14×10^{9}	1.01×10^{8}	2.10×10^{9}	1.32×10^{8}	9.07×10^{8}	3.87×10^{7}	1.70×10^{9}	1.29×10^{8}
24	3.55×10^{9}	2.13×10^{8}	1.16×10^{9}	9.63×10^{7}	2.03×10^{9}	1.41×10^{8}	8.80×10^{8}	3.88×10^{7}	1.69×10^{9}	1.27×10^{8}

Comment: SDEV = standard deviation. Values marked by red color served for the growth parameters calculations

Table 4. The minimal inhibitory concentrations for *Lactobacillus* species.

LAB	S^{2-} (mM)	OD	OD SDEV	CFU	CFU SDEV	% inh.	% inh. SDEV
L. pentosus	**0**	2.334	0.0243	**6.25×10^{9}**	1.05×10^{9}	**0**	0
	**0.038**	1.976	0.0120	**4.65×10^{9}**	1.00×10^{8}	**23.72**	11.22
	**0.076**	1.558	0.0913	**3.08×10^{9}**	1.75×10^{8}	**49.86**	5.62
	**0.114**	1.353	0.1711	**2.37×10^{9}**	1.30×10^{8}	**61.34**	4.42
	**0.153**	0.922	0.0350	**1.50×10^{9}**	6.05×10^{8}	**77.06**	5.83
	**0.191**	0.293	0.0457	**1.56×10^{6}**	5.85×10^{5}	**99.98**	0.0053
L. paracasei	**0**	2.287	0.0257	**1.29×10^{9}**	2.00×10^{7}	**0**	0
	**0.286**	2.082	0.0063	**9.40×10^{8}**	1.40×10^{8}	**27.28**	9.73
	**0.572**	1.529	0.0040	**5.27×10^{8}**	3.35×10^{7}	**59.22**	1.96
	**0.858**	0.561	0.0528	**1.00×10^{8}**	8.48×10^{7}	**92.33**	6.45
	**1.145**	0.262	0.0038	**4.05×10^{6}**	3.01×10^{6}	**99.69**	0.23
	**1.431**	0.168	0.0015	**4.19×10^{5}**	3.81×10^{5}	**99.97**	0.03
L. plantarum	**0**	2.418	0.0240	**2.18×10^{9}**	3.80×10^{8}	**0**	0
	**0.160**	2.048	0.0410	**1.48×10^{9}**	1.00×10^{8}	**30.81**	7.47
	**0.266**	1.699	0.0738	**1.01×10^{9}**	1.96×10^{8}	**53.62**	0.88
	**0.373**	0.596	0.1075	**3.02×10^{8}**	8.20×10^{7}	**86.39**	1.39
	**0.479**	0.137	0.0538	**2.15×10^{8}**	8.70×10^{7}	**90.55**	2.34
	**0.586**	0.066	0.0150	**2.13×10^{5}**	6.30×10^{4}	**99.99**	0.0012
L. fermentum	**0**	2.004	0.006	**2.00×10^{9}**	3.75×10^{8}	**0**	0
	**0.160**	1.957	0.014	**1.70×10^{9}**	3.00×10^{8}	**14.60**	1.015
	**0.266**	1.943	0.012	**1.59×10^{9}**	3.50×10^{8}	**20.80**	2.66
	**0.373**	1.883	0.037	**1.10×10^{9}**	1.00×10^{8}	**43.82**	5.55
	**0.479**	0.466	0.082	**1.34×10^{8}**	3.65×10^{7}	**92.71**	3.20
	**0.586**	0.038	0.032	**1.13×10^{6}**	9.94×10^{5}	**99.93**	0.063
L. reuteri	**0**	2.126	0.031	**3.31×10^{9}**	3.60×10^{8}	**0**	0.

	**0.286**	1.945	0.005	**2.30×10^{9}**	3.60×10^{8}	**30.88**	3.36
	**0.572**	1.715	0.061	**1.51×10^{9}**	1.00×10^{7}	**53.80**	5.33
	**0.858**	0.805	0.040	**2.26×10^{8}**	5.95×10^{7}	**92.91**	2.57
	**1.145**	0.162	0.058	**1.26×10^{8}**	125×10^{8}	**95.73**	4.24
	**1.431**	0.006	0.004	**2.70×10^{6}**	2.30×10^{6}	**99.91**	0.079

Comment: Sulfide concentration (S^{2-}); optical density (OD); standard deviation (SDEV); percentage of inhibition (% inh)

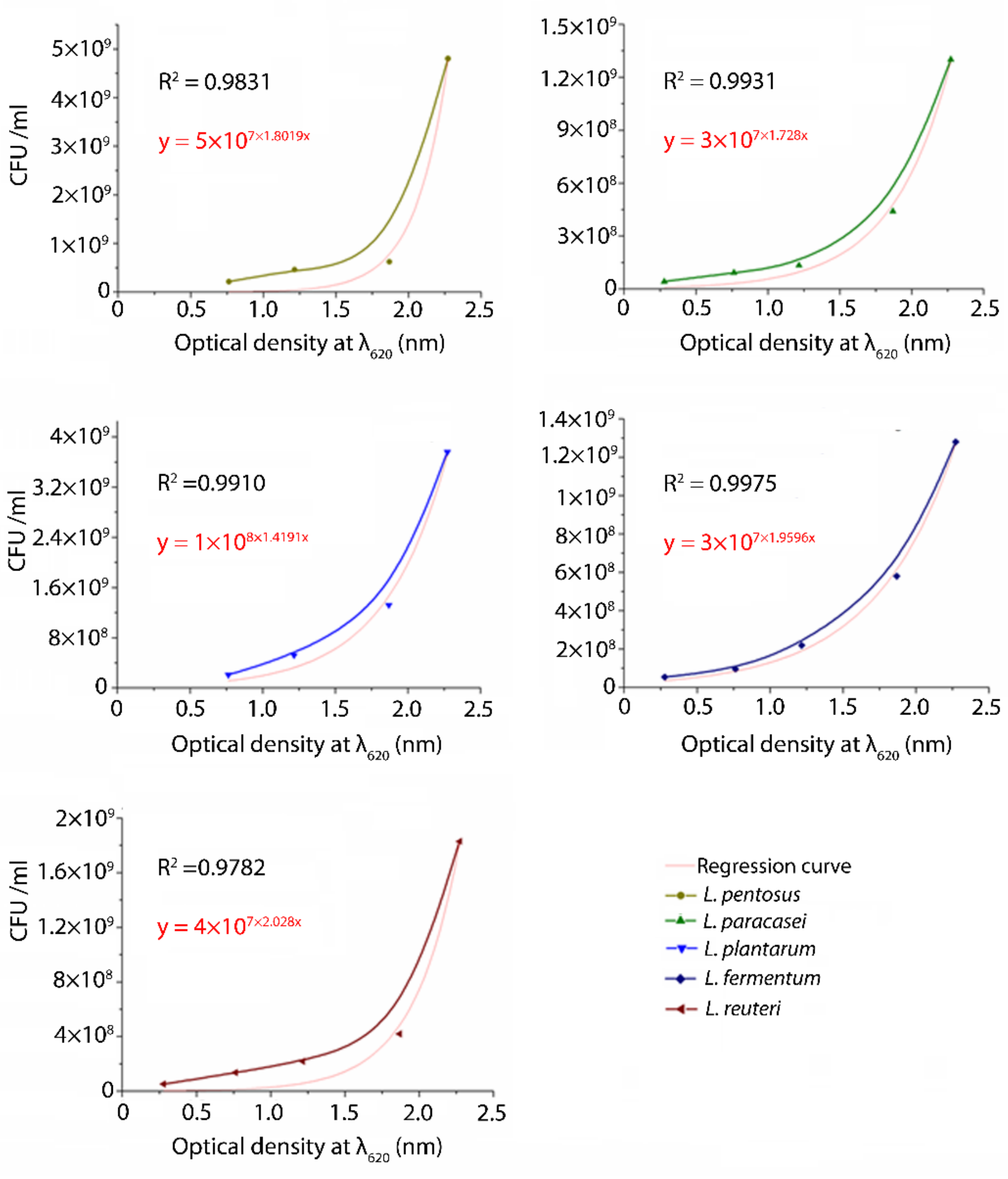

Figure 4S. The calibration curves used for the conversion of CFU to OD
